# Comparing Class II MHC DRB3 Diversity in Colombian Simmental and Simbrah Cattle Across Worldwide Bovine Populations

**DOI:** 10.3389/fgene.2022.772885

**Published:** 2022-02-04

**Authors:** Diego Ordoñez, Michel David Bohórquez, Catalina Avendaño, Manuel Alfonso Patarroyo

**Affiliations:** ^1^ Animal Science Faculty, Universidad de Ciencias Aplicadas y Ambientales (U.D.C.A), Bogotá, Colombia; ^2^ PhD Program in Tropical Health and Development, Universidad de Salamanca, Salamanca, Spain; ^3^ Molecular Biology and Immunology Department, Fundación Instituto de Inmunología de Colombia (FIDIC), Bogotá, Colombia; ^4^ MSc Program in Microbiology, Universidad Nacional de Colombia, Bogotá, Colombia; ^5^ Health Sciences Division, Main Campus, Universidad Santo Tomás, Bogotá, Colombia; ^6^ Microbiology Department, Faculty of Medicine, Universidad Nacional de Colombia, Bogotá, Colombia

**Keywords:** MHC, BoLA-DRB3, genetic diversity, peptide-binding region, genetic resistance, cattle

## Abstract

The major histocompatibility complex (MHC) exerts great influence on responses to infectious diseases and vaccination due to its fundamental role in the adaptive immune system. Knowledge about MHC polymorphism distribution among breeds can provide insights into cattle evolution and diversification as well as population-based immune response variability, thus guiding further studies. Colombian Simmental and Simbrah cattle’s *BoLA-DRB3* genetic diversity was compared to that of taurine and zebuine breeds worldwide to estimate functional diversity. High allele richness was observed for Simmental and Simbrah cattle; nevertheless, high homozygosity was associated with individual low sequence variability in both the β1 domain and the peptide binding region (PBR), thereby implying reduced MHC-presented peptide repertoire size. There were strong signals of positive selection acting on *BoLA-DRB3* in all populations, some of which were poorly structured and displayed common alleles accounting for their high genetic similarity. PBR sequence correlation analysis suggested that, except for a few populations exhibiting some divergence at PBR, global diversity regarding potential MHC-presented peptide repertoire could be similar for the cattle populations analyzed here, which points to the retention of functional diversity in spite of the selective pressures imposed by breeding.

## Introduction

The major histocompatibility complex (MHC) plays an important effector role in the adaptive immune response (Rock et al., 2016). This particular system offers a unique opportunity for addressing functional and evolutionary diversity issues in many species (Trtkova et al., 1995). The MHC is formed by a group of loci encoding specific cell surface glycoproteins which are necessary for T-lymphocyte antigen peptide recognition (Rock et al., 2016). MHC class I proteins are expressed by all nucleated cells and are related to presenting antigens to CD8^+^ T-lymphocytes processed in the intracellular compartment, thereby eliciting cytotoxic responses (Neefjes et al., 2011; Rock et al., 2016). By contrast, MHC class II proteins are expressed by professional antigen-presenting cells and are associated with the presentation of extracellular antigen peptides to CD4^+^ T-lymphocytes, triggering cellular or humoral responses against various pathogens (Neefjes et al., 2011; Rock et al., 2016).

The MHC in cattle has been called the bovine leukocyte antigen (BoLA) and the genes encoding the expression of class II antigen presentation (DR and DQ)-related molecules are located in chromosome 23 *IIa* subregion (Ellis, 2004). BoLA-DR consists of the *DRA* monomorphic locus and three *DRB* loci, of which *BoLA-DRB3*—characterized by a high degree of polymorphism, with 330 different alleles reported to date—is the only one which has been described as functional (Maccari et al., 2017). Such polymorphisms are concentrated in the second exon which encodes the peptide binding region (PBR) β1 domain and has been used for determining *BoLA-DRB3* alleles (Sigurdardóttir et al., 1991). Such high variability determines the amino acids (aa) forming PBR binding pockets, influencing the peptides presented on MHC for different alleles and setting different repertoires modulating the immune response (Ellis, 2004; Baxter et al., 2009). *BoLA-DRB3* diversity could be used for estimating potential peptide-binding repertoire size, based on the assumption that highly divergent alleles are associated with broader peptide spectra (Klein et al., 2007).

Assessing MHC genetic variability is of interest for animal science researchers, breeders, and evolutionary biologists. Variations regarding susceptibility to infectious diseases (Dietz et al., 1997; Acosta-Rodríguez et al., 2005; Martinez et al., 2006; Nascimento et al., 2006; Kulberg et al., 2007; Juliarena et al., 2008; Nikbakht Brujeni et al., 2016; Carignano et al., 2017), vaccine response (Bacon and Witter, 1995; García-Briones et al., 2000; Rupp et al., 2007; Baxter et al., 2009; Gowane et al., 2013) and production traits (Sharif et al., 1999; Rupp et al., 2007) have been associated with different *BoLA-DRB3* alleles, and information regarding their frequency has thus been used for running infectious disease control programs (Maillard et al., 2003). MHC variability patterns reflect evolutionary processes such as adaptation, selection (natural, sexual or artificial) and genetic diversity within and between populations (Trtkova et al., 1995; Edwards and Hedrick, 1998; Uinuk-Ool et al., 2002; Ujvari and Belov, 2011). Some studies have shown that decreased MHC variability (often higher rates than of neutral loci (Sutton et al., 2011)) might be caused by population bottlenecks (Bollmer et al., 2011; Mason et al., 2011; Babik et al., 2012; Taylor et al., 2012; Zhang et al., 2016), while others have shown that a high level of diversity could be maintained by pathogen-driven balancing selection or other mechanisms, despite extreme population decline (Mikko et al., 1997; Edwards and Hedrick, 1998; Garrigan and Hedrick, 2001; Aguilar et al., 2004; Borg et al., 2011; Galaverni et al., 2013; Moutou et al., 2013; Newhouse and Balakrishnan, 2015). Such information can be used for prioritizing other types of study, such as immunopeptidomic or binding affinity assays used for developing peptide-MHC *in silico* interaction predictive algorithms that could provide feedback regarding immune system response and evolution (Nielsen et al., 2008; Nielsen et al., 2018).

The Simmental is a cattle (*Bos taurus*) breed that was selected in North America and Europe, mainly for increasing meat production efficiency (Amaya et al., 2020). The introduction of Simmental to Colombia 5 decades ago aimed to increase both milk and beef production by artificial insemination-based genetic improvement schemes using semen from proven bulls in North America and Europe (Amaya et al., 2020). Simbrah is considered a composite breed developed to combine Brahman cattle (*Bos indicus*) adaptability, maternal instinct, hardiness and disease resistance with Simmental fertility, milk production and beef quality (Goszczynski et al., 2018; Amaya et al., 2020). Most tropical countries where Simbrah cattle occur have chosen a different breeding strategy, producing animals with different percentages of Zebuine genes, ranging from 1/4 (25%) to 5/8 (62.5%) based on the requirement of particular features, such as better adaptation to humid environments (Agung et al., 2016).

Despite recent advances in exploring *BoLA-DRB3* genetic diversity in cattle, a significant amount of breeds and crossbreeds still remain uncharacterized (Takeshima et al., 2003; Giovambattista et al., 2013; Takeshima et al., 2014; Takeshima et al., 2015; Takeshima et al., 2018). The aim of this study, therefore, was to describe for the first time *BoLA-DRB3* genetic diversity in the Colombian Simmental breed and its common zebuine cross, Simbrah, comparing it with that of worldwide taurine and zebuine breeds to assess the impact on potential peptide-binding repertoire size and divergence. Such new MHC diversity information will assist in introducing appropriate breeding schemes, guiding further MHC studies.

## Materials and Methods

### Study Population and DNA Extraction

Whole blood was collected from the coccygeal or jugular veins of 130 Simmental (N = 67; 5 farms) and Simbrah cattle (N = 60; 5 farms) (Supplementary Data S1), stored in EDTA-containing vacutainer tubes. Bovines were selected from extensive production systems from Colombia’s main breeding regions characterized by a reduced number of purebred animals per farm. The herds and purebred animals analyzed were randomly sampled avoiding related individuals. Genomic DNA (gDNA) was extracted using the PureLink Genomic DNA Mini Kit (Invitrogen, Carlsbad, CA, United States) and following the manufacturer’s instructions. Previous allelic richness data (Greenbaum et al., 2014) of 14 taurine and zebuine populations from Asia, South America and Europe were included for comparison (Table 1). This study was carried out following the protocol approved by the *Universidad de Ciencias Aplicadas y Ambientales*’ (U.D.C.A) Animal Research Ethics Committee (minutes No.201901).

**TABLE 1:** General information regarding the 16 cattle populations analyzed in this study.

Breed	Acronym	N	Farms/places	Country	Type	Reference
Simmental	SmtCo	67	5	Colombia	taurine	This study
Simbrah	SbhCo	60	5	Colombia	taurine	This study
Normande	NorCo	111	14	Colombia	taurine	Bohórquez et al. (2020)
Morucha	MorSp	54	15	Spain	taurine	Bohórquez et al. (2020)
Nellore	NeBo	116	2	Bolivia	zebuine	Takeshima et al. (2018)
Nellore x Brahman	NeBrPe	195	1	Peru	zebuine	Takeshima et al. (2018)
Holstein	HolAr	413	4	Argentina	taurine	Takeshima et al. (2015)
Holstein	HolBo	153	2	Bolivia	taurine	Takeshima et al. (2015)
Holstein	HolPa	127	5	Paraguay	taurine	Takeshima et al. (2015)
Holstein	HolPe	132	2	Peru	taurine	Takeshima et al. (2015)
Holstein	HolCh	113	5	Chile	taurine	Takeshima et al. (2015)
Yacumeño	YacBo	100	4	Bolivia	taurine	Giovambattista et al. (2013)
Holstein	HolJa	101	Random collection	Japan	taurine	Takeshima et al. (2003)
Brahman	BrPh	233	2	Philippines	zebuine	Takeshima et al. (2014)
Native x Brahman	NaBrPh	131	4	Philippines	zebuine	Takeshima et al. (2014)
Native	NaPh	480	4	Philippines	zebuine	Takeshima et al. (2014)

### DNA Amplification and Sequencing

*BoLA-DRB3* exon 2 was amplified with primers DRB3F (5′-TCC​CGC​ATT​GGT​GGG​TGT-3′) and DRB3R (5′-CTC​CAC​ACT​GGC​CGT​CCA​C-3′) (Ledwidge et al., 2001). The PCR mixture contained 1X Pfx amplification buffer, 300 mM of each dNTP, 0.45 mM of each primer, 1 mM MgSO_4_, 1 U Platinum Pfx DNA Polymerase (Invitrogen) and 50 ng gDNA, in a 50 ml final volume. Two independent reactions were performed for each sample, following previous recommendations to avoid chimeric product formation (Lenz and Becker, 2008). The thermal profile consisted of a denaturation step at 94°C for 5 min followed by 30 cycles of 94°C for 30 s, 64°C for 30 s and 68°C for 1 min, with no final extension. Wizard SV Gel and PCR Clean-Up System (Promega, Madison, WI, United States) were used for purifying the amplicons according to the manufacturer’s instructions prior to sequencing both directions using the BigDye Terminator Kit.

### Sequence Data Analysis

CLC Main Workbench (CLC bio, Aarhus, Denmark) was used for assembling and editing each sequence independently. Polymorphic positions were recognized for producing a final consensus sequence containing IUPAC ambiguity codes. HAPLOFINDER (Miltiadou et al., 2003) was used for assigning each animal’s genotype by comparing the obtained sequences to the *BoLA-DRB3* allele sequences reported in the IPD-MHC database (Maccari et al., 2017), as previously indicated (Baxter et al., 2008).

### Sequence Diversity, Hardy-Weinberg Equilibrium and Selection Signatures

The number of alleles (N_a_) and allele frequencies were manually obtained by direct counting using the maximum likelihood method for estimating standard errors for allele frequencies according to Li (Li, 1976). Nei and Chesser’s method (Nei, 1978) was used for calculating the observed heterozygosity (*h*_*o*_) and ARLEQUIN v.3.5 (Excoffier and Lischer, 2010) for estimating the unbiased expected heterozygosity (*h*_*e*_). The correlation between sample size and these estimators was used for assessing their dependence. Allele richness (a measure of the average amount of alleles per locus) was also used for comparing the number of alleles found in each population independently from sample size (Greenbaum et al., 2014). The *F*_*IS*_ index (Weir and Cockerham, 1984) was estimated for determining potential departures from Hardy-Weinberg equilibrium using the exact test of significance implemented in GENEPOP v.4.5.0 (Rousset, 2008). GENEDOC v.2.7 (Nicholas, 1997) was used for calculating identity and similarity percentages (as assessed by the BLOSUM62 substitution matrix) for all genotypes observed within populations for the whole β1 domain and PBR positions [considering the previously reported 31 putative positions constituting the MHC-DRB PBR (Suárez et al., 2006)]. PBR sequence logos, along with PBR sequence correlation between populations, were analyzed to further evaluate differences in potential MHC-presented peptide repertoire among cattle populations. MEGA X (Kumar et al., 2018) was used for calculating the average amount of synonymous (*d*_S_) and nonsynonymous (*d*_N_) substitutions per site by Nei-Gojobori’s method with Jukes-Cantor correction. The Z-test was used for assessing *d*_N_/*d*_S_ ratio significance. Codons subject to positive selection were inferred using maximum likelihood (FEL (Kosakovsky Pond and Frost, 2005)) and Bayesian (MEME (Murrell et al., 2012)) methods using the Datamonkey web server (Delport et al., 2010), using a *p*-value <0.1 as significant threshold.

### Population Structure, Genetic Differentiation and PBR Similarity Correlation

Population structure and genetic differentiations between populations were evaluated by calculating pairwise *F*_*ST*_ statistics (Weir and Cockerham, 1984) using ARLEQUIN. POPTREE v.2 (Takezaki et al., 2010) was used for calculating genetic distances (*D*_*A*_) (Nei, 1978) from allele frequency data and PAST v.3.2 (Hammer et al., 2001) for generating an allele frequency-based principal component analysis (PCA). WebLogo web server (Crooks et al., 2004) was used to construct a logo for PBR positions for each population using BLOSUM62 substitution matrix for each position including all alleles observed within populations. A 620-coordinate vector (31 positions x 20 possible aa genotypes) representing each PBR population was used for calculating Pearson correlation coefficients using R package *amap* (Ihaka and Gentleman, 1996), while R package *ape* was used to construct an UPGMA dendrogram (Paradis et al., 2004). Mitochondrial DNA D-loop sequences from individuals of several of the populations analyzed here were recovered from GenBank (Supplementary Data S2) and used for evaluating MHC allele distribution based on genetic affinity between populations. A maximum likelihood phylogeny was built using Tamura-3-parameter model (best fit model) for mitochondrial DNA and JTT model for *BoLA-DRB3* aa sequences using MEGA X.

## Results

### *BoLA-DRB3* Allele Distribution in Colombian Simmental and Simbrah Cattle

Sixty *BoLA-DRB3* alleles were identified in both focal populations: 37 in Simmental and 43 in Simbrah cattle (GenBank accession numbers OM100952-OM101010). No new alleles were detected. Three alleles (BoLA-DRB3*002:01, *005:03* and *012:01*) occurred with >5% frequency in both populations and seven (BoLA-DRB3*005:01, *008:01*, *010:01*, *013:01*, *015:01*, *016:01* and *022:01*) only in the Simmental breed. Four alleles (BoLA-DRB3*002:01, *005:03*, *012:01* and *022:01*) occurred with >5% frequency in Simbrah, accounting for 31.67% of cumulated frequency, whereas 10 alleles (BoLA-DRB3*002:01, *005:01*, *005:03*, *008:01*, *010:01*, *012:01*, *013:01*, *015:01*, *016:01* and *030:01*) with >5% frequency, accounting for 61.94% cumulative frequency, occurred in Simmental. Twenty alleles were shared by both cattle populations, accounting for 64.7% of their mean cumulative allele frequency (summation of weighted mean allele frequency for all shared alleles) (Table2).

**TABLE 2:** Allele frequency and standard error for *BoLA-DRB3* alleles common to Simmental and Simbrah cattle.

Allele	Simbrah	Simmental
001:01	0.008 ± 0.008	0.037 ± 0.016
002:01	**0.058 ± 0.021**	**0.082 ± 0.023**
003:01	0.017 ± 0.011	0.022 ± 0.012
005:01	0.025 ± 0.014	**0.06 ± 0.02**
005:03	**0.058 ± 0.021**	**0.06 ± 0.02**
009:01	0.017 ± 0.011	0.022 ± 0.012
010:01	0.033 ± 0.016	**0.06 ± 0.02**
011:01	0.025 ± 0.014	0.022 ± 0.012
012:01	**0.117 ± 0.029**	**0.052 ± 0.019**
013:01	0.025 ± 0.014	**0.052 ± 0.019**
014:01:01	0.017 ± 0.011	0.022 ± 0.012
016:01	0.017 ± 0.011	**0.097 ± 0.025**
017:03	0.033 ± 0.016	0.007 ± 0.007
018:01	0.017 ± 0.011	0.015 ± 0.01
019:02	0.017 ± 0.011	0.007 ± 0.007
020:01:01	0.025 ± 0.014	0.007 ± 0.007
021:01	0.025 ± 0.014	0.007 ± 0.007
026:01	0.008 ± 0.008	0.037 ± 0.016
027:03	0.008 ± 0.008	0.007 ± 0.007
030:01	0.017 ± 0.011	**0.052 ± 0.019**

Alleles having >5% frequency are highlighted in bold.

### *BoLA-DRB3* Genetic Diversity, Hardy-Weinberg Equilibrium and Selection Pattern

The number of alleles corrected for sample size effect (*Rs*) showed that Simmental and Simbrah had a high genetic diversity, similar to what was observed for Colombian Normande and Philippine populations (Table 3). Sample size appeared to have little effect on both *h*_*o*_ (*r* = 0.36) and *h*_*e*_ (*r* = 0.06), highlighting these estimates as being good proxy of *BoLA-DRB3* diversity in the populations analyzed here. Colombian Simmental, Colombian Normande, Spanish Morucha and Colombian Simbrah had the lowest *h*_*o*_ and the highest *h*_*e*_ values, in contrast with the results for other breeds (Table 3; Supplementary Data S1). Consequently, the highest departures from Hardy-Weinberg equilibrium were observed in these four breeds, as evidenced by the statistically significant *F*_*IS*_ fixation index (Table 3). These results indicated significant heterozygote deficiency regarding the *BoLA-DRB3* locus in Colombian Simmental and Colombian Simbrah cattle.

**TABLE 3:** Genetic diversity estimates and Hardy-Weinberg equilibrium within cattle populations.

Population	N	N_a_	*R*_*s*_	*h*_*o*_	*h*_*e*_	*F*_*IS*_ (S.E.)	*dN*	*dS*	*dN*/*dS*
SmtCo	67	37	34	0.642	0.959	0.333 (0.0005)**	0.107	0.03	*3.57*
SbhCo	60	43	41.6	0.783	0.966	0.191 (0.0004)**	0.116	0.026	*4.46*
NorCo	111	53	38.2	0.667	0.955	0.303 (<0.0001)**	0.111	0.028	*3.96*
MorSp	54	29	29	0.667	0.913	0.271 (<0.0001)**	0.105	0.026	*4.04*
HolAr	413	31	20.5	0.833	0.908	0.082 (0.0001)**	0.115	0.025	*4.6*
HolBo	153	21	18	0.928	0.895	−0.038 (0.0054)	0.124	0.031	*4*
HolPa	127	26	19.6	0.835	0.891	0.064 (0.0140) *	0.117	0.024	*4.88*
HolPe	132	19	16.6	0.902	0.886	−0.018 (0.0151)	0.12	0.032	*3.75*
HolJa	101	18	15.7	0.922	0.902	−0.022 (0.0324)	0.115	0.027	*4.26*
HolCh	113	21	17.2	0.841	0.893	0.059 (0.0067)*	0.113	0.028	*4.04*
YacBo	100	33	30.1	0.91	0.947	0.04 (0.0399)	0.116	0.023	*5.04*
NeBrPe	195	33	23.5	0.759	0.855	0.113 (0.0015)*	0.116	0.027	*4.3*
BrPh	233	57	36.3	0.884	0.95	0.07 (0.0019)*	0.112	0.026	*4.31*
NeBo	116	26	21.9	0.784	0.87	0.099 (0.0412)	0.117	0.023	*5.09*
NaBrPh	131	56	40.8	0.908	0.966	0.06 (0.0201)	0.113	0.023	*4.91*
NaPh	480	71	36.8	0.915	0.959	0.044 (0.0040)*	0.112	0.024	*4.67*

Number of individuals (N), Number of alleles (N_a_), allelic richness (*R*_*s*_), observed (*h*_*o*_) and expected (*h*_*e*_) heterozygosity, non-synonymous/synonymous substitution ratio (*dN*/*dS*) and Hardy-Weinberg equilibrium as evaluated by *F*_*IS*_, coefficients along with standard errors (SE). Statistical significance is indicated in bold and * or ** for *p* < 0.05 and *p* < 0.001, respectively.

Whole β1 domain identity ranged from 82.45% (Philippine Brahman) to 88.95% (Colombian Normande), with Colombian Simmental (88.79%) and Simbrah (86.33%) displayed some of the highest values (Table 4). Bolivian Holstein and Colombian Normande had the lowest (87.45%) and the highest (92.5%) whole β1 domain similarity, respectively. PBR identity and similarity differences were broader than those for the whole β1 domain. The former ranged from 65.87% (Peruvian Holstein) to 80.81% (Spanish Morucha), whereas the latter ranged from 75.18% (Bolivian Holstein) to 87.59% (Spanish Morucha). Colombian Simmental and Simbrah displayed some of the highest identity and similarity values regarding both whole β1 domain and PBR.

**TABLE 4:** Genetic diversity at sequence level within cattle populations.

Population	β1 domain		PBR	
	Identity	Similarity	Identity	Similarity
SmtCo	88.79 (9.6)	92.33 (6.9)	78.67 (17.7)	84.61 (13.1)
SbhCo	86.33 (8.6)	91.2 (5.9)	73.68 (16.9)	82.48 (11.7)
NorCo	88.95 (8.8)	92.5 (6.2)	77.75 (17.4)	84.59 (12.5)
MorSp	88.35 (9.5)	92.22 (6.9)	80.81 (15.7)	87.59 (10.7)
HolAr	85.34 (7.6)	89.66 (5.7)	69.67 (16)	78.03 (12.3)
HolBo	82.71 (6.6)	87.45 (5.3)	66.31 (12.2)	75.18 (10.1)
HolPa	84.87 (7.8)	89.29 (5.7)	68.61 (16.4)	77.04 (12.5)
HolPe	83.89 (7.1)	88.77 (5.5)	65.87 (15)	75.72 (11.9)
HolJa	83.44 (6.1)	88.56 (4.5)	66.15 (12.6)	76.38 (9.2)
HolCh	84.44 (7.7)	88.83 (5.9)	67.4 (16.4)	75.91 (12.9)
YacBo	83.15 (7.2)	87.9 (5.4)	66.87 (13.7)	75.7 (11.1)
NeBrPe	85.66 (10.1)	90.36 (7.5)	73.43 (17.6)	82.62 (12.3)
BrPh	82.44 (8.1)	87.71 (6.1)	69.81 (14)	79.15 (10.1)
NeBo	85.41 (8.9)	90.85 (6.1)	71.96 (16.6)	83.58 (11)
NaBrPh	82.53 (7.5)	88.14 (5.8)	68.68 (12.9)	78.96 (9.3)
NaPh	83.13 (7.1)	88.71 (5.4)	68.96 (12.9)	79.23 (9.5)

Similarity was calculated according to BLOSUM62 substitution matrix. PBR, peptide binding region.

Average *d*_N_ was significantly higher than average *d*_S_ for Colombian Simmental and Simbrah populations, similar to what was observed across all the other populations (Table 3). Moreover, codons 10, 11, 12, 26, 30, 32, 37, 57, 70, 71, 74, 77 and 78 were identified as sites under diversifying selection, most of which (11, 12, 26, 30, 37, 57, 70, 71, 74 and 78) were PBR-related. Mitochondrial DNA clusters were mostly formed by individuals of one to four breeds. Infrequently occurring allele lineages (i.e., BoLA-DRB3*025:01:02, 025:02, 025:01:01, 037:01 and *039:01* in most populations) or moderately occurring ones (i.e., BoLA-DRB3*030:01, 030:02, 036:01, 023:01 and *044:01*) were identified in populations that are not closely related (Supplementary Data S3 and Supplementary Data S4).

### Population Structure and Genetic Differentiation Based on *BoLA-DRB3* Gene

**FIGURE 1:**
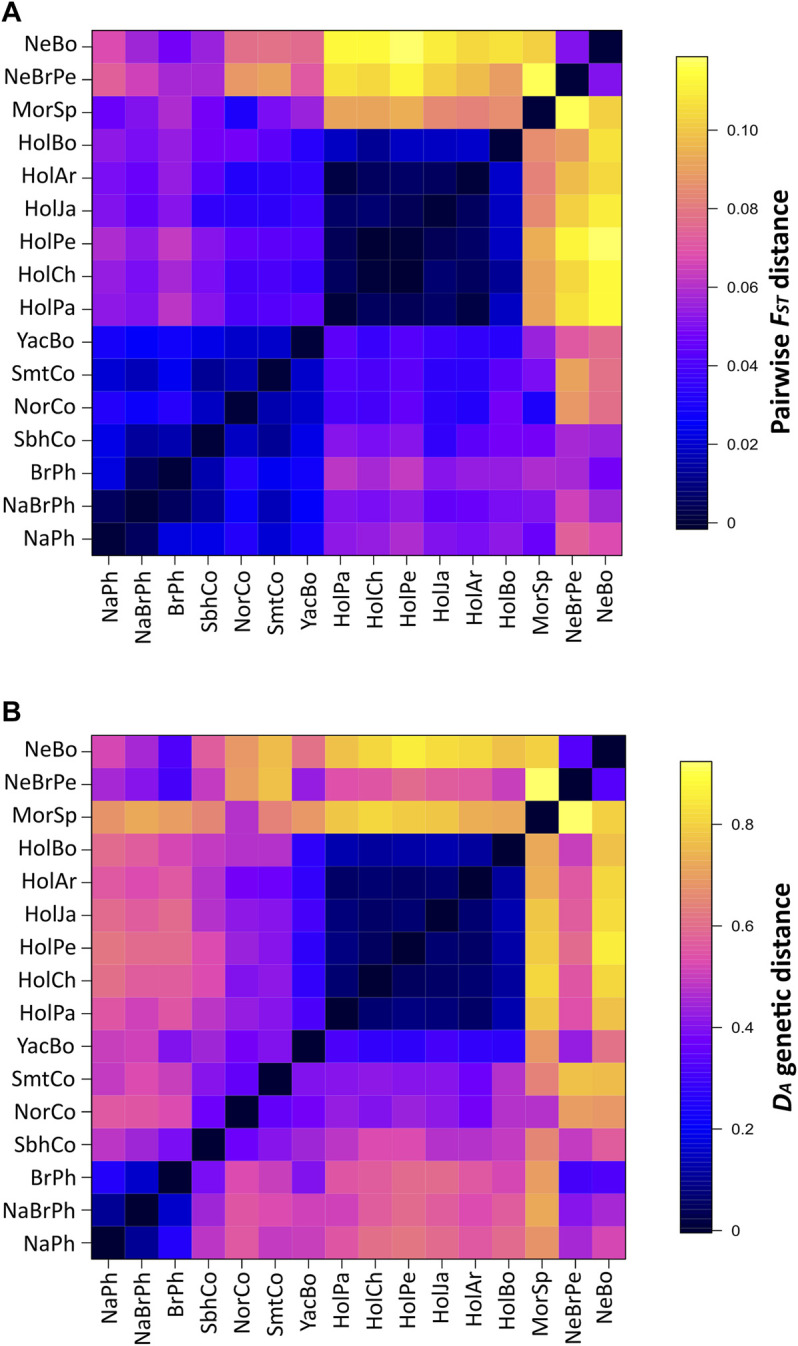
Pairwise *F*_*ST*_ and *D*_*A*_ genetic distances between populations. Symmetrical heat maps for pairwise *F*_*ST*_ values **(A)** and *D*_*A*_ genetic distance **(B)** between cattle populations based on *BoLA-DRB3* alleles. All pairwise *F*_*ST*_ comparisons (except for Chilean with Peruvian Holstein and Argentinian with Paraguayan Holstein) were significant at 0.05 level.

Pairwise *F*_*ST*_ values ranged from −0.0009 (Chilean Holstein with Peruvian Holstein) to 0.1185 (Peruvian Holstein with Bolivian Nellore) (Figure 1A: Supplementary Data S5). All comparisons were statistically significant, except for Chilean with Peruvian Holstein and Argentinian with Paraguayan Holstein. Two groups with low *F*_*ST*_ values were mainly observed (Figure 1A); the first consisted of Holstein populations (0.0088 mean *F*_*ST*_ value), while the second was formed by Philippine, Colombian Simmental, Colombian Simbrah, Bolivian Yacumeño and Colombian Normande populations (0.0208 mean *F*_*ST*_ value). Differences between Colombian Simmental and Simbrah were also observed regarding Argentinian, Peruvian, Chilean and Paraguayan Holstein.

Colombian Simbrah was differentiated from native Philippine, Colombian Normande, Bolivian Yacumeño and Holstein populations to a greater extent than Colombian Simmental. On the contrary, Colombian Simmental was more differentiated from Philippine Native-Brahman, Philippine Brahman, Spanish Morucha, Peruvian Nellore-Brahman and Bolivian Nellore than Colombian Simbrah (Figure 1A: Supplementary Data S5).

Genetic distance *D*_*A*_ clustering was similar to that based on pairwise *F*_*ST*_ among Holstein and the Philippine populations (Figure 1B). The latter group was well-differentiated from Colombian Simmental, Colombian Simbrah, Bolivian Yacumeño and Colombian Normande populations on the basis of *D*_*A*_ but not on pairwise *F*_*ST*_. Mean genetic distance values for these breeds indicated that common alleles could explain a large amount of their cumulative allele frequency. Thus, the Holstein population group (0.078 mean *D*_*A*_ distance) had 28 alleles in common, accounting for 95.2% of their mean cumulative allele frequency. Ten of these common alleles (BoLA-DRB3*001:01, *002:01*, *006:01*, *009:02*, *010:01*, *011:01*, *012:01*, *14:01:01*, *015:01* and *027:03*) occurred with >5% frequency in at least one of these populations. The Philippine group (0.169 mean *D*_*A*_ distance) had 65 common alleles accounting for 93.1% of their mean cumulative allele frequency, of which 8 (BoLA-DRB3*002:01, 003:01, 012:01, 015:01, 022:01, 030:01, 036:01 and *041:01*) were alleles with >5% frequency. The group formed by Colombian and Bolivian Yacumeño populations (0.387 mean *D*_*A*_ distance) had 49 alleles in common, accounting for 69.1% of their mean cumulative allele frequency. Seventeen (BoLA-DRB3*001:01, *002:01*, *005:01*, *005:03*, *007:01*, *008:01*, *009:02*, *010:01*, *012:01*, *013:01*, *014:01:01*, *015:01*, *016:01*, *018:01*, *022:01*, *030:01* and *048:02*) of these shared alleles had >5% frequency.

**FIGURE 2:**
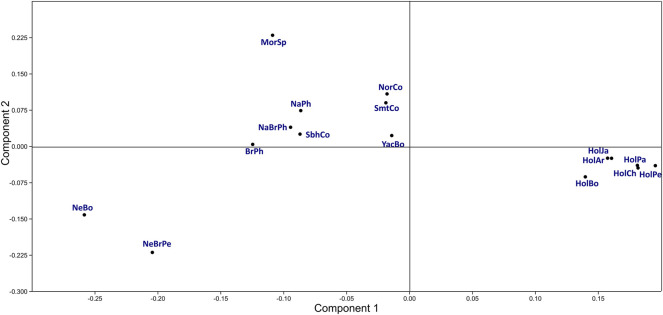
Principal component analysis using *BoLA-DRB3* allele frequencies for cattle populations.

Groups identified in the first two principal component (PC) plots were consistent with those identified in *F*_*ST*_ and *D*_*A*_ distance analysis (Figure 2: Supplementary Data S6). The first PC (42.7% variance) differentiated four main groups. The Holstein population group was characterized by high BoLA-DRB3*015:01, *011:01*, *001:01*, *027:03*, *010:01*, *012:01*, *014:01:01* and *009:02* allele frequency, while, group formed by Normande, Simmental and Yacumeño by intermediate frequency regarding the same alleles; both groups had intermediate BoLA-DRB3*006:01, *017:01*, *016:01*, *009:01* and *002:01* allele frequencies. Bolivian Nellore and Peruvian Nellore-Brahman along with a group formed by Philippine Native, Philippine Native-Brahman, Colombian Simbrah, Philippine Brahman and Spanish Morucha were at the other extreme of the first PC. These two groups were characterized by high or intermediate BoLA-DRB3*048:02, *030:01*, *028:01* and *022:01* allele frequency. Spanish Morucha was remarkably differentiated in the second PC (20.3% variance) due to high BoLA-DRB3*048:02, *003:01* and *005:01* frequency, whereas Nellore cattle had high BoLA-DRB3*028:01, *009:02* and *022:01* frequencies.

### PBR Sequence Similarity and Correlation Between Populations

**FIGURE 3:**
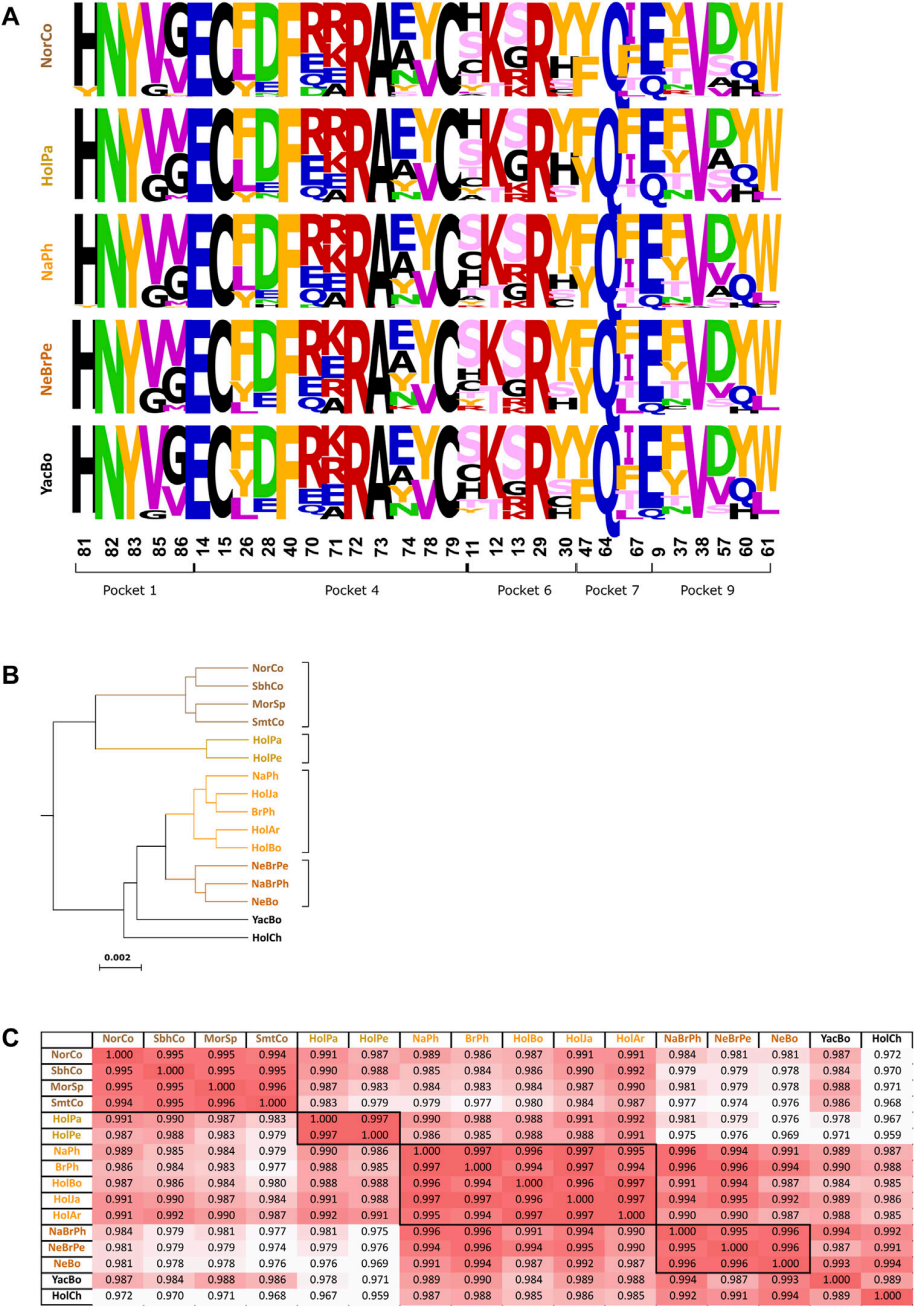
Peptide binding region similarity and correlation. **(A)** *BoLA-DRB3* allele peptide binding region (PBR) logos representative of each cattle population group. Conservative aa changes according to the BLOSUM 62 substitution matrix are shown in the same color; different colors indicate non-conservative changes: FYW (yellow), MILV (purple), RK (red), EQ (blue), DN (green), ST (pink), HCPAG (black). PBR Pockets and their aa forming positions are indicated below the logos. **(B)** Clustering analysis based on correlation analysis. **(C)** Pearson correlation coefficient matrix for populations based on PBR sequence variability. Population group colors are the same for panels **(A,B,C)**.

PBR logo representation showed that positions 70, 71, 74, 11, 13, 30, 67, 37, and 57 were highly variable and tended to accumulate non-conservative changes, while positions 82, 83, 14, 15, 40, 72, 73, 79, 29, 64, 47, 9, and 38 were invariable or only displayed conservative changes (Figure 3A). Some highly variable sites were found under positive selection (11, 30, 37, 57, 70, 71, and 74). PBR logos had very similar substitution patterns for all populations. Likewise, Pearson correlation coefficients (PCC) were remarkably high, having high global correlation (PCC = 0.987), thereby indicating low variation for aa frequency for each PBR position among all populations.

PBR correlation analysis identified four major groups (Figures 3B,C). The first one consisted of Colombian Normande, Colombian Simbrah, Spanish Morucha and Colombian Simmental (0.994 mean PCC). It is worth mentioning that the most notable differences between Colombian Simmental and Simbrah occurred regarding aa frequency for positions 70, 30 and 57. Paraguayan Holstein and Peruvian Holstein clustered together (PCC = 0.996). The third group was formed by native Philippine, Philippine Brahman, Bolivian Holstein, Japanese Holstein and Argentinian Holstein (0.996 mean PCC). The fourth group included native Philippine-Brahman, Peruvian Nellore-Brahman and Bolivian Nellore (0.995 mean PCC). Divergence between population groups was mainly due to PBR Pockets 6 (PCC = 0.981), 7 (PCC = 0.979) and 9 (PCC = 0.978), while Pockets 1 (PCC = 0.992) and 4 (PCC = 0.992) were less variable.

## Discussion

The MHC influences susceptibility and resistance to infectious diseases, vaccine responses and productions traits. MHC allele distribution information can be used to guide resource-consumption studies (such as immunopeptidomic or binding affinity assays) aimed at identifying MHC-associated peptides and developing *in silico* binding predictive algorithms that can be used for understanding and predicting immune response patterns (Nielsen et al., 2018; Rappazzo et al., 2020). MHC high polymorphism can also provide insights into populations evolutionary history. Nevertheless, MHC diversity has only been explored in a few cattle breeds to date (Takeshima et al., 2003; Giovambattista et al., 2013; Takeshima et al., 2014; Takeshima et al., 2015; Takeshima et al., 2018). In this study, we have first characterized *BoLA-DRB3* genetic diversity in the taurine Simmental breed and in its most common cross with zebuine cattle in tropical regions, the Simbrah in Colombia. Considering the recent origin of Colombian Simmental and Simbrah cattle, a large percentage of highly related animals is expected since small herds are derived from few parents, thus reflecting a potentially reduced genetic diversity, a condition previously found for other pure cattle breeds in the country (Amaya et al., 2020; Bohórquez et al., 2020).

Cattle populations varied considerably in terms of allele richness and Hardy-Weinberg equilibrium. It is worth noting that unequal sample size have no significant impact on genetic diversity estimates such as *h*_*o*_ (unbiased parametric value estimator, mainly determined by the sampling method used (Nei and Chesser, 1983; Bohórquez et al., 2020)), *h*_*e*_ or *R*_*s*_, also when the pertinent corrections were applied (Nei, 1978; Leberg, 2002). Simmental and Simbrah *R*_*s*_ and *h*_*e*_ were among the highest values, similar to those of Normande and Philippine populations. However, differences in *R*_*s*_ (or N_a_) associated with similar *h*_*e*_ values across populations (as observed for Simmental and Simbrah) indicated a marked allele frequency distribution variation, while *h*_*o*_ values were the lowest for Colombian cattle, with *F*_*IS*_ indices being thereby the highest for these populations. Population sub-structuring may have reduced *h*_*o*_ [by means of the Wahlund effect, i.e., reduced observed heterozygosity in a population caused by subpopulation structure (De Meeûs, 2017)], thus magnifying allele frequency differences compared to those found in other populations.

Natural selection and random genetic drift (notoriously exerting higher effects in populations with smaller effective population size) are factors affecting allele frequency distribution (Nei and Tajima, 1981; Tajima and Nei, 1984; Akashi et al., 2012; Husemann et al., 2016), which in the case of the MHC is expected to reflect balancing selection with heterozygote excess (Hughes and Nei, 1988; Hughes and Yeager, 1998; Takeshima et al., 2008). Even though *F*_*IS*_ indicated heterozygote deficiency for *BoLA-DRB3*, the persistence of identical or similar alleles (allelic lineages) in spite of the overall genetic differentiation points to the action of balancing selection. Moreover, the highly significant *d*_N_/*d*_S_ values and codons identified under positive selection suggest that aa variability preference has not been eroded in these cattle. Although drift and non-random mating are well-known factors leading to increased homozygosity (Crow, 2010) as well as small effective population sizes and inbreeding, the use of only one locus hampered from singling out the underlying cause of such pattern. Likewise, the high *F*_*IS*_ values for some of these populations, indicating higher homozygote percentages than those expected for Hardy-Weinberg equilibrium, points to the occurrence of evolutionary forces acting on these populations. Therefore, further studies based on genome-wide data including both neutral and non-neutral loci, are necessary to get a comprehensive picture of the evolutionary forces acting on this system, and of their relative contributions [in Simmental, selection based on production and its small effective population size should be taken into account (Amaya et al., 2020; de Araujo Neto et al., 2020)].

Alleles fell into different categories based on their distribution throughout the populations tested. The first category consisted of alleles widely distributed in populations from different continents and often displaying relatively high frequencies. These alleles, such as BoLA-DRB3*011:01, are possibly present in all populations, or absent in a few of them, such as BoLA-DRB3*010:01, *012:01* and *014:01:01*. Considering that taurine and zebuine cattle were domesticated in more than two independent events (Beja-Pereira et al., 2006; Decker et al., 2014), these alleles probably predate *Bos primigenius* divergence which gave rise to these cattle types. Such alleles might have been either present in just a subset of the founder populations or ubiquitous before undergoing secondary loss due to random genetic drift and/or natural selection (alternatively, their very low frequencies impaired their sampling) (Barton, 1996; Sutton et al., 2011). Another category consisted of alleles, such as BoLA-DRB3*017:01, *006:01*, *009:01* and *027:10*, found predominantly in taurine or zebuine cattle with their presence in the other type of cattle populations being possibly indicative of admixture. The last category includes the alleles found exclusively in some populations and displaying low frequencies, possibly representing the most recently arisen ones (Uinuk-Ool et al., 2002). Despite forming just a moderate proportion of known *BoLA-DRB3* alleles found in and Simbrah cattle (67 and 60 out of 330, respectively) (Maccari et al., 2017), they occur with significant frequency in other cattle populations, representing the major allele variants (Bohórquez et al., 2020) (Supplementary Data S3, Supplementary Data S4 and Supplementary Data S6). Nevertheless, some alleles contributing towards the distinction of Colombian Simmental from Simbrah were also significant in differentiating zebuine from taurine cattle, such as BoLA-DRB3*015:01 and *022:01* frequently occurring in taurine and zebuine cattle, respectively (Takeshima et al., 2018). Alleles private to Simmental or Simbrah further contributed to their differentiation.

Although most of the target bovine populations analyzed here were genetically well-differentiated based on *BoLA-DRB3*, others had a shallow structure due to the sharing of several alleles occurring with high or intermediate frequency. These populations clustered into five groups according to the measures of differentiation used. Low mean distance values indicated high genetic affinity for these populations and more detailed analysis showed that commonly occurring alleles accounted for a large percentage of their mean cumulative allele frequency. Several factors may result in weak structure. For instance, the limited genetic distance between Colombian Simmental and Normande might be due to sample origin, as geographical dispersal patterns in cattle reflects those of exportation and co-migration in humans (Decker et al., 2014), as well as similar selection pressure (Bohórquez et al., 2020). Furthermore, as zebuine introgression occurred independently in American and Indian cattle (Decker et al., 2014), crossbreeding with Brahman may have led to low genetic differentiation between Simbrah and Philippine populations. These results contrasted with the weak differentiation in Holstein cattle, such breed forming a very compact group in spite of multiple sample origins (Takeshima et al., 2015), possibly as the result of intense selective pressure regarding milk production traits (Bohórquez et al., 2020) and a high level of gene flow *via* genetic improvement strategies, thereby leading to a high degree of homogenization (Spieth, 1974; Leroy et al., 2013).

MHC PBR positions hosted the highest β1 domain variability associated with the peptides to which an allele could bind (Hughes and Nei, 1989; Stern et al., 1994). The similarity/identity matrix of the MHC-DRB PBR position across cattle populations suggested potential differences in MHC-presented peptide repertoire size (Table 4). Colombian populations had the highest identity and similarity values in both β1 domain and PBR. This could be due to high overall homozygosity but also to a limited variability at these specific loci and suggested that these animals had smaller individual MHC-presented peptide repertoires. Nevertheless, the good correlation regarding PBR aa sequence and logo analysis may suggest that potential population-related MHC-presented peptide repertoire diversity could be equivalent among all cattle populations analyzed here, with just a few groups displaying a much higher variability. This implies that breeding is unlikely to have decreased functional MHC variability, which bears important implications for peptide-based vaccine design, so that different cattle populations could be targeted using similar peptide combinations. Although it has been shown that decreased MHC variability might be caused by population bottlenecks (Bollmer et al., 2011; Zhang et al., 2016), balancing selection driven by pathogens can still maintain a high degree of diversity (Aguilar et al., 2004; Newhouse and Balakrishnan, 2015). Moutou *et al.*, have shown that functional polymorphism may be lower than genetic polymorphism in pigs (Moutou et al., 2013). The highest similarities within and between porcine populations were mainly due to high correlation in PBR Pocket 1 and 4, whereas a higher divergence was observed for Pocket 6, 7 and 9. This could have arisen from Pocket 1, 4 and 6 aa preferences as anchor positions (Rappazzo et al., 2020). This work has shown that, in spite of high values for some genetic diversity measures regarding *BoLA-DRB3* (such as allele richness and expected heterozygosity), Colombian Simmental, Simbrah and other cattle populations may have a limited potential MHC-presented peptide repertoire diversity could be similar among all cattle populations analyzed and that breeding did not decrease functional diversity. Additional analyses directly addressing peptide repertoire diversity are needed to confirm these results.

## Data Availability

The original contributions presented in the study are included in the article/Supplementary Material, further inquiries can be directed to the corresponding author.
