# Supplementary material for: Comparing Class II MHC DRB3 Diversity in Colombian Simmental and Simbrah Cattle Across Worldwide Bovine Populations
Source: Front Genet. 2022 Feb 4;13:772885. doi: 10.3389/fgene.2022.772885 (PMC8854852; doi:10.3389/fgene.2022.772885)
Supplement: Supplementary file 1 [file DataSheet2.PDF]

Supplementary Data S2. D-loop GenBank accession numbers of cattle from different breeds used for evaluating MHC allele distribution based on genetic affinity.

Cattle breed	GenBank accession number
Bolivian Yacumeño	N382548, JN382550- JN382561
Philippine Brahman	AB079324, AB079326, AB079327
Holstein	AB003799
Native Philippine	AB079301-AB079325
Nelore	AY235791,AY235792,AY235795,AY235797-AY235802
Simmental	FN562583, FN562611, FN562612
Spanish Morucha	DQ515550-DQ515552
