# Supplementary material for: Comparing Class II MHC DRB3 Diversity in Colombian Simmental and Simbrah Cattle Across Worldwide Bovine Populations
Source: Front Genet. 2022 Feb 4;13:772885. doi: 10.3389/fgene.2022.772885 (PMC8854852; doi:10.3389/fgene.2022.772885)
Supplement: Supplementary file 2 [file DataSheet4.PDF]

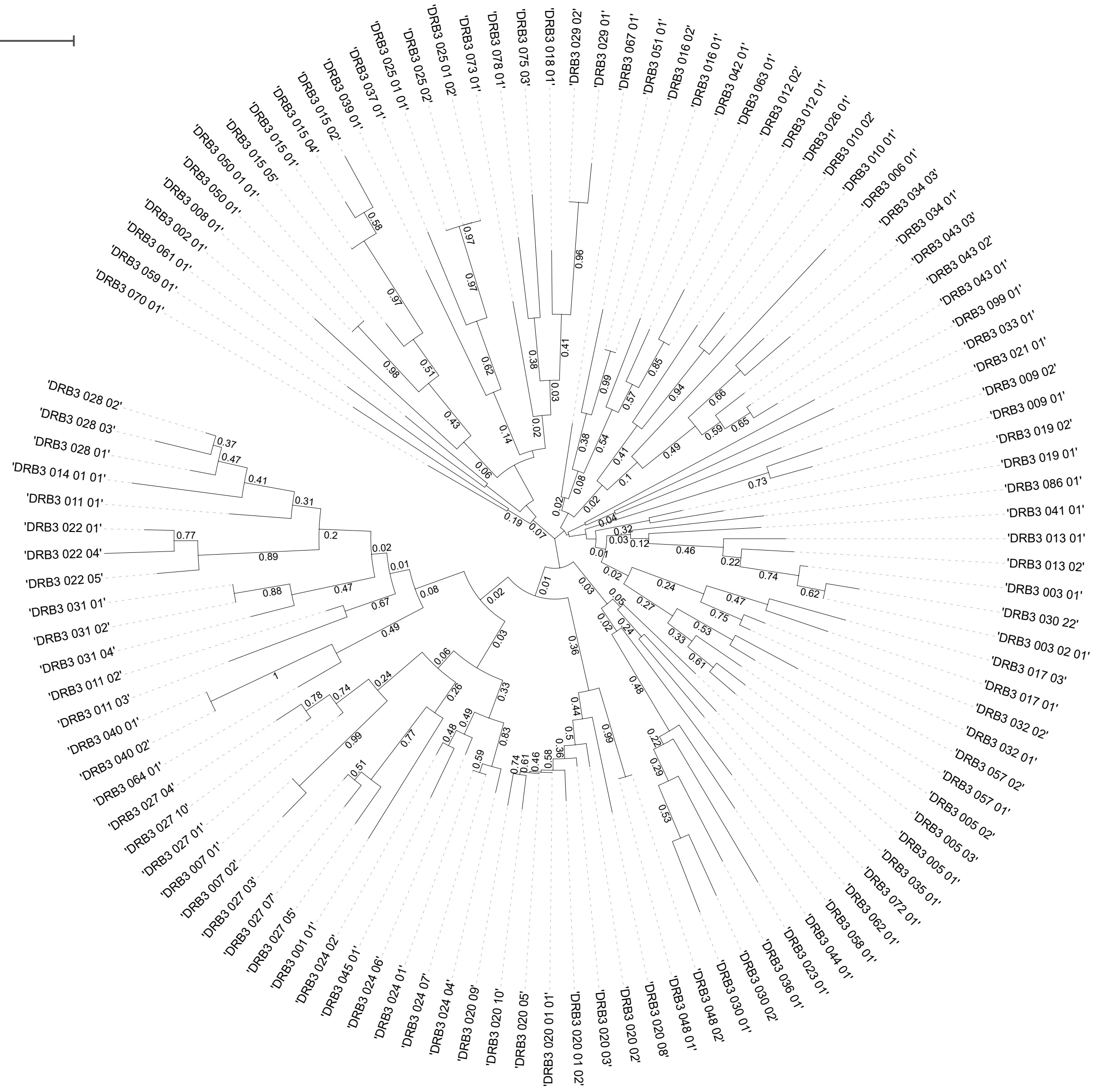

Supplementary Data S4. Maximum likelihood *BoLA-DRB3* phylogeny of amino acid sequences based on the JTT model and 1000 bootstrap replicates.
