# Supplementary material for: Comparing Class II MHC DRB3 Diversity in Colombian Simmental and Simbrah Cattle Across Worldwide Bovine Populations
Source: Front Genet. 2022 Feb 4;13:772885. doi: 10.3389/fgene.2022.772885 (PMC8854852; doi:10.3389/fgene.2022.772885)
Supplement: Supplementary file 3 [file DataSheet6.PDF]

Supplementary Data S6. Allele frequencies and first two principal component loadings for each allele and cattle population. Color intensity is proportional to allele frequency.

NeBrPe	NeBo	BrPh	NaBrPh	SbhCo	NaPh	MorSp	NorCo	SmtCo	YacBo	HolBo	HolAr	HolJa	HolPa	HolCh	HolPe	Allele	PC 1	PC 2
0.01795	0	0.02146	0.0458	0	0.05312	0	0	0.05224	0.05	0.18627	0.14891	0.13235	0.17717	0.21681	0.21591	015:01	0.49982	-0.22533
0.00513	0.00431	0.00215	0.01527	0.025	0.00937	0.00926	0.03153	0.02239	0.035	0.11765	0.11622	0.16667	0.16535	0.11947	0.17045	011:01	0.40931	-0.15206
0.00256	0	0	0.00382	0.00833	0.00104	0	0.1036	0.03731	0.02	0.05882	0.18039	0.11275	0.17717	0.12389	0.11364	001:01	0.3791	-0.080316
0.03846	0	0	0.00382	0.00833	0.00313	0	0.0045	0.00746	0.02	0.04902	0.07748	0.08824	0.05906	0.04867	0.05303	027:03	0.15939	-0.12489
0	0.02586	0.02361	0	0.03333	0.00313	0.02778	0.06306	0.0597	0.035	0.10458	0.05085	0.05882	0.06299	0.0885	0.06061	010:01	0.15766	-0.0068542
0.00769	0	0.07296	0.04962	0.11667	0.0125	0.02778	0.05405	0.05224	0.015	0.04902	0.07385	0.14216	0.05118	0.07965	0.09848	012:01	0.15669	0.025296
0.01538	0.00431	0.01931	0.00763	0.01667	0.00625	0	0.07658	0.02239	0.07	0.01634	0.03995	0.04902	0.01969	0.09292	0.08333	014:01:01	0.12241	-0.013676
0.15897	0.01293	0.03433	0.00763	0.01667	0.00625	0	0	0	0.095	0.16013	0.06538	0.05882	0.07087	0.07965	0.05682	009:02	0.10843	-0.34397
0.00256	0	0	0	0.01667	0	0.00926	0.01351	0	0.035	0.10784	0.01695	0.04412	0.00394	0.01327	0.00379	006:01	0.074531	-0.030791
0.01282	0	0	0	0.00833	0.00104	0	0.00901	0	0.02	0.01307	0.02542	0.0098	0.02756	0.0177	0.0303	017:01	0.052684	-0.036583
0.00256	0.01724	0.00858	0.01145	0.01667	0.025	0	0.01802	0.09701	0.04	0.00327	0.03511	0.03922	0.01181	0.01327	0.01894	016:01	0.021093	0.047529
0	0	0	0.00382	0.01667	0.00313	0	0.0045	0.02239	0.045	0	0.0109	0.0098	0.01181	0.00885	0.01136	009:01	0.018006	0.015852
0.01795	0	0.05794	0.08015	0.05833	0.08333	0	0.06757	0.08209	0.07	0.02614	0.03269	0.05392	0.06693	0.0354	0.02273	002:01	0.017708	0.091982
0	0	0	0	0	0	0	0	0	0	0	0.01574	0	0	0	0.01136	045:01	0.014025	-0.0052153
0.00256	0.00431	0.02575	0.00763	0.01667	0.00208	0.01852	0.01351	0.01493	0.09	0.03268	0.023	0	0.01181	0.00885	0.01894	018:01	0.013774	0.029585
0	0	0.00215	0.00763	0	0.00208	0	0.03153	0.05224	0	0.00327	0.01816	0	0.00787	0.00442	0	008:01	0.0084345	0.046812
0.01538	0	0.01931	0.01145	0	0.02187	0.00926	0.04505	0.01493	0.12	0.01307	0.01937	0.0098	0.00394	0.02212	0.01136	007:01	0.0031999	0.042382
0	0	0.00429	0.00763	0	0.00104	0	0.00901	0.00746	0	0	0.00242	0	0	0.00885	0	020:02	0.0010138	0.010034
0	0	0	0	0	0	0	0.0045	0	0	0	0	0	0	0	0	024:07	-0.00024069	0.0030684
0	0	0	0	0	0	0	0.0045	0	0	0	0	0	0	0	0	028:03	-0.00024069	0.0030684
0	0	0	0	0	0	0	0.0045	0	0	0	0	0	0	0	0	031:04	-0.00024069	0.0030684
0	0	0	0	0	0	0	0.0045	0	0	0	0	0	0	0	0	075:03	-0.00024069	0.0030684
0	0	0	0	0	0	0	0	0.00746	0	0	0	0	0	0	0	015:04	-0.00041957	0.0042261
0	0	0	0	0	0	0	0	0.00746	0	0	0	0	0	0	0	059:01	-0.00041957	0.0042261
0	0	0	0	0	0	0	0	0.00746	0	0	0	0	0	0	0	067:01	-0.00041957	0.0042261
0	0	0	0	0	0	0	0	0	0.01	0	0	0	0	0	0	029:02	-0.00042356	0.0014164
0	0	0	0	0	0.00104	0	0	0	0.005	0	0	0	0	0	0	025:02	-0.0004804	0.0011921
0	0	0	0	0	0.00208	0	0	0	0	0	0	0	0	0	0	048:01	-0.00053723	0.00096774
0	0	0	0	0	0	0	0.01351	0	0	0	0	0	0	0	0	070:01	-0.0007226	0.009212
0	0	0	0	0	0.00104	0	0.00901	0	0	0	0	0	0	0	0	005:02	-0.00075053	0.0066275
0	0	0.00215	0	0	0	0	0	0	0	0	0	0	0	0	0	030:02	-0.00080063	0.58766
0	0	0.00215	0	0	0	0	0	0	0	0	0	0	0	0	0	031:02	-0.00080063	0.58766
0	0	0.00215	0	0	0	0	0	0	0	0	0	0	0	0	0	061:01	-0.00080063	0.58766
0	0	0	0.00382	0	0.00104	0	0	0	0	0	0	0	0	0	0	015:02	-0.0013476	0.0014354
0	0	0	0	0	0	0	0.01802	0.00746	0	0	0	0	0	0	0	015:05	-0.0013834	0.016513
0	0	0	0	0	0	0	0.02703	0	0	0	0	0	0	0	0	024:06	-0.0014457	0.018431
0	0	0	0	0	0	0	0.0045	0.02239	0	0	0	0	0	0	0	051:01	-0.0015	0.015752
0	0	0	0.00382	0	0.00208	0	0	0	0	0	0	0	0	0	0	062:01	-0.0016162	0.0019193
0	0	0	0	0	0.00313	0	0	0.01493	0	0	0	0	0	0	0	063:01	-0.0016481	0.0099141
0	0	0.00215	0	0	0.00104	0	0.0045	0.00746	0	0	0	0	0	0	0	027:01	-0.0017295	0.0078371
0	0	0	0	0.00833	0	0	0	0	0	0	0	0	0	0	0	086:01	-0.0021662	0.0013338
0	0	0	0	0.00833	0	0	0	0	0	0	0	0	0	0	0	099:01	-0.0021662	0.0013338
0	0	0	0	0.00833	0	0	0.0045	0	0	0	0	0	0	0	0	022:05	-0.0024069	0.0044022
0	0	0.00429	0	0	0.00417	0	0	0	0	0	0	0	0	0	0	020:05	-0.0026746	0.0020574
0	0	0.00429	0.00382	0	0	0	0	0	0	0	0	0	0	0	0	064:01	-0.0026765	0.0010688
0	0	0	0	0.00833	0	0	0.01351	0	0	0	0	0	0	0	0	007:02	-0.0028888	0.010546
0	0	0	0.00763	0	0.00313	0	0	0	0	0	0	0	0	0	0	025:01:02	-0.0029636	0.0033569
0	0	0	0	0	0	0.00926	0	0	0	0	0	0	0	0	0	012:02	-0.0030112	0.013361
0	0	0	0	0	0	0.00926	0	0	0	0	0	0	0	0	0	020:10	-0.0030112	0.013361
0	0	0	0	0	0	0.00926	0	0	0	0	0	0	0	0	0	030:22	-0.0030112	0.013361
0	0	0	0	0	0	0.00926	0	0	0	0	0	0	0	0	0	078:01	-0.0030112	0.013361
0	0	0	0	0.00833	0	0	0.01802	0	0	0	0	0	0	0	0	011:02	-0.00313	0.013621
0.00513	0	0	0	0	0	0	0	0	0	0	0	0	0	0	0	057:01	-0.0031323	-0.0070523
0	0	0.00429	0	0	0.00625	0	0	0	0	0	0	0	0	0	0	043:03	-0.0032118	0.0030251
0	0	0.00644	0	0	0.00313	0	0	0	0.005	0	0	0	0	0	0	039:01	-0.0034184	0.0023405
0	0	0	0	0	0	0.00926	0	0.00746	0	0	0	0	0	0	0	024:01	-0.0034308	0.017588
0	0	0.00429	0.00382	0	0.00313	0	0	0	0	0	0	0	0	0	0	027:04	-0.003485	0.0025251
0	0	0	0.00763	0	0.00937	0	0	0	0	0	0	0	0	0	0	073:01	-0.0045753	0.0062601
0	0	0	0	0.01667	0	0	0.0045	0	0	0	0	0	0	0	0	022:04	-0.0045756	0.0057376
0	0	0	0	0.01667	0	0	0.0045	0	0	0	0	0	0	0	0	029:01	-0.0045756	0.0057376
0	0	0.00644	0.00382	0	0.00625	0	0	0	0	0	0	0	0	0	0	020:08	-0.0050914	0.0040355
0	0	0.00429	0.01145	0	0.00208	0	0	0	0	0	0	0	0	0	0	016:02	-0.0053689	0.0039372
0	0	0	0.00382	0	0.00104	0.01852	0.01351	0	0	0	0	0	0	0.00442	0	032:01	-0.0056957	0.036137
0	0	0	0	0	0	0.01852	0	0	0	0	0	0	0	0	0	020:09	-0.0060224	0.026723
0	0	0	0	0	0	0.01852	0	0	0	0	0	0	0	0	0	024:02	-0.0060224	0.026723
0	0	0	0	0	0	0.01852	0	0	0	0	0	0	0	0	0	024:04	-0.0060224	0.026723
0	0	0	0.00382	0.01667	0	0	0.0045	0.00746	0	0	0	0	0	0	0	019:02	-0.0060742	0.010915
0	0	0.00644	0	0.01667	0.00313	0	0.0045	0	0	0	0.00242	0	0	0	0	027:07	-0.0066449	0.0070027
0	0.00431	0	0.00763	0.01667	0.00521	0	0	0	0	0	0	0	0.00787	0	0	034:01	-0.0069072	0.0012395
0.00769	0	0.01717	0	0	0.00104	0	0	0	0	0	0	0	0.00787	0	0	027:05	-0.0071021	-0.011548
0	0	0	0.00382	0	0.02604	0	0	0	0	0	0	0	0	0	0	050:01	-0.0078047	0.013067
0	0	0.00429	0.00763	0	0.01979	0	0	0	0	0	0	0	0	0	0	072:01	-0.0088641	0.011225
0	0	0	0	0.03333	0	0	0.0045	0	0	0	0	0	0	0	0	040:01	-0.008908	0.0084053
0	0	0.00429	0	0.025	0.00104	0	0	0.00746	0.005	0	0	0	0	0	0	021:01	-0.0089987	0.0095385
0	0	0	0	0	0	0.02778	0	0	0	0	0	0	0	0	0	058:01	-0.0090336	0.040084
0	0	0	0	0	0	0.02778	0.0045	0.01493	0	0	0	0	0	0	0	010:02	-0.010114	0.051611
0.01026	0	0	0	0	0	0.01852	0	0.01493	0.01	0	0.00121	0	0	0	0.00379	037:01	-0.010771	0.021366
0	0	0	0	0.025	0.00104	0	0.00901	0.05224	0.015	0	0	0	0	0	0	013:01	-0.010825	0.042349
0.00769	0	0.01073	0.00382	0	0.00417	0	0.0045	0	0	0	0	0	0	0	0	020:03	-0.011088	-0.0043183
0.00256	0	0.00215	0.00763	0.025	0.00208	0.03704	0.01351	0	0.005	0	0.00121	0.0098	0.00787	0.00442	0	020:01:02	-0.012616	0.061925
0	0	0.00215	0.0229	0	0.02292	0	0	0	0	0	0	0	0	0	0	040:02	-0.013189	0.016427
0	0	0	0.0229	0	0.0375	0	0	0	0	0	0	0	0	0	0	050:01:01	-0.016154	0.023152
0	0	0.00429	0.0229	0	0.03333	0	0.0045	0	0	0	0	0	0	0	0	034:03	-0.016915	0.024397
0.00513	0.00862	0.00644	0.01527	0	0.00417	0	0	0	0	0	0	0	0	0	0	042:01	-0.017575	-0.0087813
0	0.01724	0.01073	0.01145	0	0.00208	0	0.0045	0	0.01	0	0.00121	0	0.00394	0	0	028:02	-0.019041	-0.0078495
0	0	0.00215	0.03053	0.01667	0.01979	0	0.00901	0	0	0	0	0	0	0	0	043:02	-0.019352	0.025684
0	0.01724	0.01288	0.00763	0	0.00417	0.00926	0	0.01493	0.005	0	0.00121	0.0049	0.00394	0	0	032:02	-0.020352	0.0095206
0	0	0.00429	0.00763	0.00833	0.00313	0.0463	0.0045	0	0	0.00327	0	0	0	0	0	023:01	-0.02066	0.073394
0	0	0	0	0.05833	0.01042	0	0.0045	0.0597	0	0	0	0	0	0	0	005:03	-0.021458	0.051076
0.01282	0	0.01288	0.01145	0.03333	0.00104	0	0	0	0	0	0.00242	0	0.00394	0	0	019:01	-0.021526	-0.0099324
0	0.02586	0.00215	0.00763	0	0.00104	0	0	0.00746	0	0	0	0	0	0	0	013:02	-0.023607	-0.016277
0.00513	0.01293	0	0.01908	0	0.02396	0	0	0	0	0	0	0	0	0	0	011:03	-0.024692	-0.0026252
0	0	0	0	0.025	0	0.05556	0.01351	0.00746	0	0	0.00121	0	0	0	0	020:01:01	-0.025142	0.097426
0	0	0.00858	0.03817	0	0.05208	0	0	0	0	0	0	0	0	0	0	041:01	-0.027428	0.033973
0.00769	0.00862	0.01502	0.00763	0	0.00937	0.01852	0	0	0	0	0	0	0	0	0	003:02:01	-0.027541	0.015173
0	0.00862	0.01288	0.01145	0	0.03125	0.01852	0	0.00746	0	0	0	0	0	0	0	043:01	-0.029198	0.041044
0	0.01293	0.00429	0.00763	0.01667	0.00729	0.0463	0.0045	0	0	0.00327	0.00726	0	0	0	0	044:01	-0.030473	0.06409
0.01026	0.02155	0.02146	0.00763	0	0.01146	0	0	0	0.025	0	0	0	0	0	0	033:01	-0.037066	-0.021867
0	0	0.00429	0	0.025	0.00729	0.11111	0.05405	0.0597	0.045	0.01961	0.00121	0	0	0	0.01136	005:01	-0.038891	0.23414
0.00513	0	0.04721	0.02672	0.03333	0.01458	0	0	0.00746	0	0	0.00121	0	0	0	0	017:03	-0.040544	0.017057
0	0.03879	0.00858	0	0.00833	0	0.00926	0.01351	0.03731	0	0	0	0	0	0	0	026:01	-0.041139	0.010858
0.02821	0	0.01502	0.03817	0	0.04167	0	0.0045	0	0	0	0	0	0	0	0	025:01:01	-0.044603	-0.0064065
0.07949	0.00862	0.00429	0	0	0.01354	0	0	0	0.02	0	0	0	0	0	0	027:10	-0.061132	-0.10768
0	0	0.00858	0.04962	0.01667	0.11667	0.12037	0.00901	0.02239	0	0.00327	0.02058	0.0049	0.01969	0	0	003:01	-0.068529	0.25207
0.01538	0.05603	0.01931	0.01145	0.03333	0.01979	0	0	0	0.01	0	0	0	0	0	0	035:01	-0.077272	-0.05152
0.05128	0.03879	0.01502	0.0229	0.00833	0.04583	0	0	0	0	0	0	0	0	0	0	057:02	-0.087321	-0.076144
0.01795	0.07328	0.03219	0.03817	0	0.01354	0	0.00901	0	0	0	0	0	0	0.00442	0	031:01	-0.091882	-0.068103
0.06154	0.02155	0.08584	0.06107	0.00833	0.025	0	0	0	0.01	0.01961	0.00121	0	0.00394	0	0.00379	036:01	-0.099383	-0.081606
0	0.02586	0.01073	0.00382	0	0.00521	0.23148	0.0991	0	0.015	0	0	0	0	0	0	048:02	-0.10759	0.38443
0.02051	0.05172	0.13948	0.08779	0.01667	0.03958	0	0	0.05224	0.01	0	0	0	0	0	0	030:01	-0.14711	0.0036866
0.02564	0.24569	0.04506	0.0229	0.01667	0.01354	0	0.00901	0	0.015	0	0	0	0.00787	0	0	028:01	-0.23327	-0.23102
0.32051	0.23276	0.06438	0.03817	0.08333	0.02083	0	0.01351	0	0.02	0.0098	0.00484	0.0049	0.00394	0.00442	0	022:01	-0.42552	-0.60835
