# Supplementary material for: Comparing Class II MHC DRB3 Diversity in Colombian Simmental and Simbrah Cattle Across Worldwide Bovine Populations
Source: Front Genet. 2022 Feb 4;13:772885. doi: 10.3389/fgene.2022.772885 (PMC8854852; doi:10.3389/fgene.2022.772885)

Supplementary Data S3. *BoLA-DRB3* allele frequency distribution and mitochondrial DNA phylogeny. (A) Maximum likelihood D-loop phylogeny based on Tamura-3-parameter model and 1000 bootstrap replicates. GenBank accession numbers are shown. (B) *BoLA-DRB3* allele phylogeny inferred using aa sequences and the JTT model. Color intensity is proportional to allele frequency.

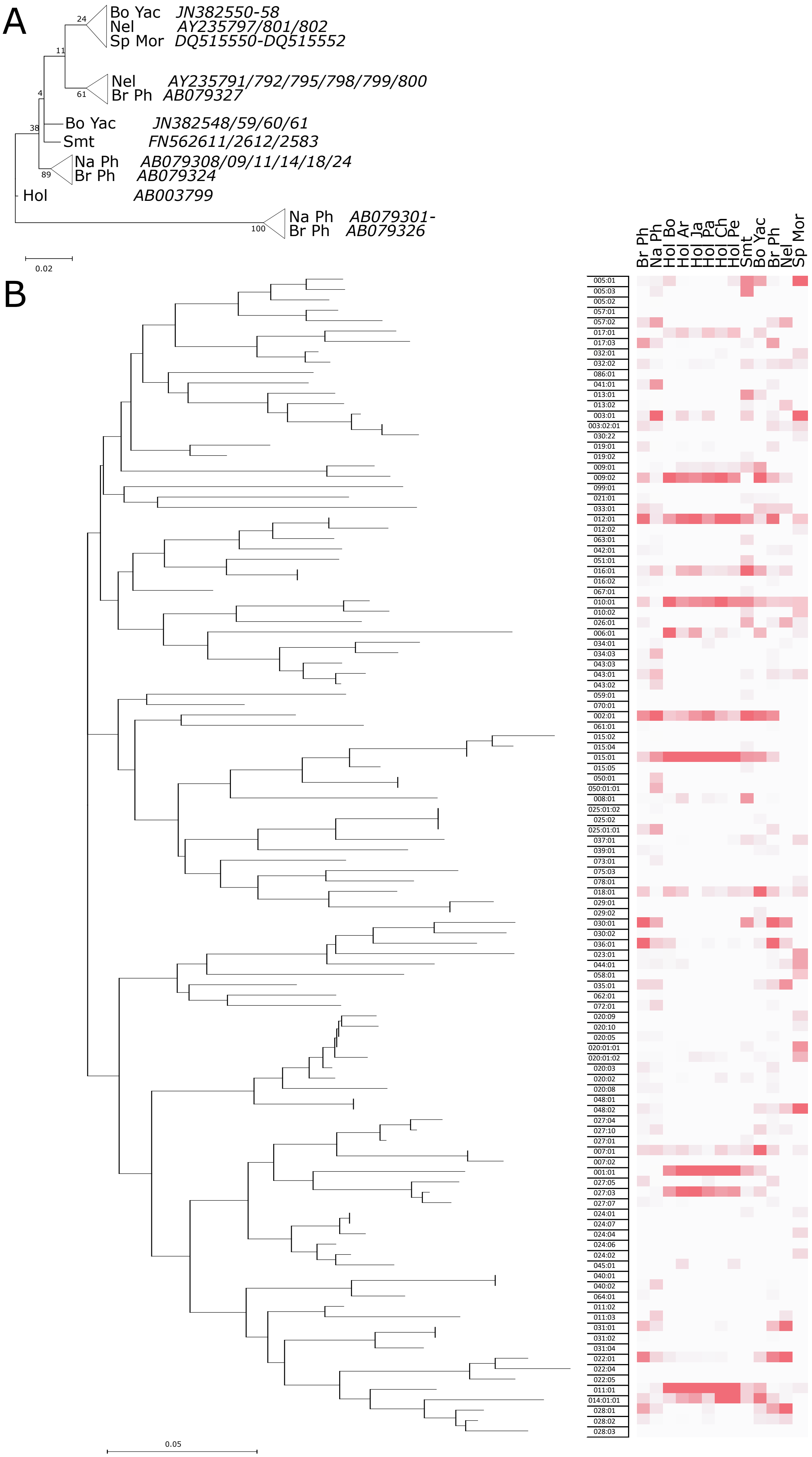

Supplement: Supplementary file 4 [file DataSheet3.PDF]
