# Supplementary material for: Comparing Class II MHC DRB3 Diversity in Colombian Simmental and Simbrah Cattle Across Worldwide Bovine Populations
Source: Front Genet. 2022 Feb 4;13:772885. doi: 10.3389/fgene.2022.772885 (PMC8854852; doi:10.3389/fgene.2022.772885)
Supplement: Supplementary file 5 [file DataSheet1.PDF]

Supplementary Data S1. Genotypes and sampling locality of Simmental (Smt) and Simbrah (Sbh) cattle. *BoLA-DRB3* alleles, Colombian department (Dpt) and municipality (Mun), breed, and individual identifier (ID) are indicated.

Dpt	Mun	Breed	Number	ID	Allele 1	Allele 2
Cundinamarca	Tocancipá	Smt	1	11	009:01	011:01
		Smt	2	191	009:01	009:01
		Smt	3	25	012:01	012:01
		Smt	4	1862	002:01	002:01
		Smt	5	179	016:01	016:01
		Smt	6	114	005:03	026:01
		Smt	7	116	005:01	002:01
		Smt	8	12	005:01	026:01
		Smt	9	23	013:01	026:01
		Smt	10	jamaica13	010:01	015:01
		Smt	11	1	030:01	030:01
		Smt	12	037-9	001:01	007:01
		Smt	13	14	005:03	012:01
		Smt	14	177	018:01	002:01
	Sesquilé	Smt	15	11--4	012:01	008:01
		Smt	16	C1	003:01	013:01
		Smt	17	1115	013:01	026:01
		Smt	18	1015	030:01	030:01
		Smt	19	1118	001:01	037:01
		Smt	20	aw1	015:01	043:01
		Smt	21	br7	012:01	012:01
		Smt	22	372	005:01	002:01
		Smt	23	10	010:01	010:01
		Smt	24	177	008:01	067:01
	Sutatausa	Smt	25	7352	013:01	013:02
		Smt	26	2	014:01:01	027:01
		Smt	27	11	051:01	059:01
		Smt	28	20	005:01	005:01
		Smt	29	123	002:01	002:01
		Smt	30	182	005:01	005:01
		Smt	31	202	002:01	020:02
		Smt	32	210	051:01	015:04
		Smt	33	230	016:01	015:01
		Smt	34	402	013:01	063:01
		Smt	35	403	016:01	016:01
		Smt	36	1440	016:01	016:01
		Smt	37	1453	026:01	063:01
		Smt	38	102	003:01	013:01

	Sopo	Smt	39	c72	016:01	016:01
		Smt	40	387	008:01	008:01
		Smt	41	C71	002:01	011:01
		Smt	42	280	001:01	017:03
		Smt	43	67	005:03	005:03
		Smt	44	73	015:01	024:01
		Smt	45	c44	005:03	027:03
		Smt	46	c391	032:02	032:02
		Smt	47	c74	010:02	011:01
		Smt	48	86	014:01:01	014:01:01
		Smt	49	70	021:01	015:05
		Smt	50	5944	005:03	005:03
		Smt	51	2528	007:01	037:01
Boyacá	Guateque	Smt	52	G1	010:01	019:02
		Smt	53	G2	010:01	010:01
		Smt	54	G3	008:01	008:01
		Smt	55	G4	016:01	016:01
		Smt	56	G5	002:01	002:01
		Smt	57	G6	005:01	030:01
		Smt	58	G7	001:01	015:01
		Smt	59	G8	001:01	012:01
		Smt	61	G10	010:01	010:01
		Smt	62	G11	005:03	015:01
		Smt	64	G13	010:02	051:01
		Smt	65	G14	016:01	008:01
		Smt	66	G15	003:01	030:01
		Smt	67	G16	015:01	030:01
		Smt	68	G17	016:01	018:01
		Smt	70	G19	013:01	020:01:01
Antioquia	Medellin	Sbh	1	7	010:01	012:01
		Sbh	2	8	012:01	017:03
		Sbh	3	13	003:01	020:01:01
		Sbh	4	15	022:01	022:01
		Sbh	5	16	006:01	006:01
		Sbh	6	20	010:01	010:01
		Sbh	7	23	022:01	022:01
		Sbh	8	24	009:01	012:01
		Sbh	9	2	020:01:02	007:02
		Sbh	10	3	022:01	022:05
		Sbh	11	6	026:01	005:03
		Sbh	12	18	012:01	027:07
		Sbh	13	19	005:01	035:01
		Sbh	14	14	016:01	016:01
		Sbh	15	5	002:01	002:01

		Sbh	16	10	001:01	012:01
		Sbh	17	21	044:01	044:01
		Sbh	18	22	012:01	019:02
		Sbh	19	11	010:01	027:07
		Sbh	20	17	012:01	034:01
		Sbh	21	12	005:01	035:01
		Sbh	22	9	021:01	043:02
Cundinamarca	Puerto Salgar	Sbh	23	11	027:03	019:02
		Sbh	24	19	029:01	022:04
		Sbh	25	7	012:01	022:01
		Sbh	26	17	029:01	022:04
		Sbh	27	2	005:01	005:03
		Sbh	28	9	040:01	040:01
		Sbh	29	18	011:01	005:03
		Sbh	30	8	012:01	034:01
		Sbh	31	13	021:01	043:02
		Sbh	32	11a	019:01	019:01
		Sbh	33	4	009:02	011:02
		Sbh	34	16	020:01:02	005:03
		Sbh	35	1	030:01	030:01
		Sbh	36	15	021:01	040:01
Meta	Pompeya-acacias	Sbh	37	2	020:01:02	005:03
		Sbh	38	68	023:01	002:01
		Sbh	39	71	011:01	005:03
		Sbh	40	6	002:01	002:01
		Sbh	41	4	086:01	099:01
		Sbh	42	5	013:01	020:01:01
		Sbh	43	10	014:01:01	013:01
		Sbh	44	19	018:01	022:01
		Sbh	45	3	017:03	035:01
		Sbh	46	1	012:01	035:01
		Sbh	47	6a	005:03	012:01
		Sbh	48	44	057:02	009:02
		Sbh	49	62	012:01	017:03
		Sbh	50	8	036:01	040:01
		Sbh	51	69	002:01	014:01:01
		Sbh	52	87	013:01	019:01
		Sbh	53	c73	002:01	019:01
		Sbh	54	15	012:01	017:03
		Sbh	55	13	022:01	022:01
		Sbh	56	16	009:01	012:01
		Sbh	57	74	011:01	017:01
		Sbh	58	7	028:01	028:01
		Sbh	59	24	022:01	018:01

		Sbh	60	23	003:01	020:01:01
