# Supplementary material for: Comparing Class II MHC DRB3 Diversity in Colombian Simmental and Simbrah Cattle Across Worldwide Bovine Populations
Source: Front Genet. 2022 Feb 4;13:772885. doi: 10.3389/fgene.2022.772885 (PMC8854852; doi:10.3389/fgene.2022.772885)
Supplement: Supplementary file 6 [file DataSheet5.PDF]

Supplementary Data S5. Pairwise F_{ST} and D_A genetic distance matrix values.

Asymmetric matrices are shown with values for F_{ST} (top) and D_A (bottom) below the diagonal.

F_{ST} values matrix

	NaPh	NaBrPh	BrPh	SbhCo	NorCo	SmtCo	YacBo	HolPa	HolCh	HolPe	HolJa	HolAr	HolBo	MorSp	NeBrPe	NelBo
NaPh	0															
NaBrPh	0.0051	0														
BrPh	0.02243	0.00521	0													
SbhCo	0.02264	0.01348	0.01558	0												
NorCo	0.03117	0.02642	0.03224	0.01859	0											
SmtCo	0.02122	0.01741	0.02394	0.01264	0.01526	0										
YacBo	0.0305	0.02739	0.02959	0.02496	0.02025	0.02053	0									
HolPa	0.05333	0.05014	0.06178	0.05144	0.04022	0.04172	0.04378	0								
HolCh	0.05454	0.04995	0.05835	0.04927	0.03908	0.04004	0.0369	0.00467	0							
HolPe	0.05889	0.05373	0.063	0.05205	0.04488	0.04425	0.04335	0.00469	-0.00096	0						
HolJa	0.05121	0.04403	0.05241	0.03566	0.03438	0.03444	0.03813	0.00661	0.00681	0.00436	0					
HolAr	0.04954	0.04583	0.05542	0.04344	0.03194	0.03455	0.03598	0.00163	0.00539	0.00675	0.00542	0				
HolBo	0.05602	0.05205	0.05729	0.05005	0.05047	0.04645	0.03269	0.0189	0.01253	0.01779	0.01793	0.02017	0			
MorSp	0.04584	0.05048	0.05979	0.04799	0.03043	0.04979	0.05674	0.09143	0.09161	0.09437	0.08484	0.08237	0.08773	0		
NeBrPe	0.07329	0.06527	0.05787	0.05811	0.08759	0.09021	0.07026	0.10711	0.10462	0.11244	0.10295	0.09823	0.09097	0.11723	0	
NelBo	0.06777	0.05666	0.04865	0.05548	0.07659	0.07879	0.07791	0.11312	0.11314	0.1185	0.10928	0.10557	0.10995	0.10201	0.05111	0

D_A values matrix

	NaPh	NaBrPh	BrPh	SbhCo	NorCo	SmtCo	YacBo	HolPa	HolCh	HolPe	HolJa	HolAr	HolBo	MorSp	NeBrPe	NelBo
NaPh	0															
NaBrPh	0.098	0														
BrPh	0.246	0.164	0													
SbhCo	0.481	0.446	0.388	0												
NorCo	0.558	0.549	0.527	0.372	0											
SmtCo	0.495	0.528	0.5	0.41	0.351	0										
YacBo	0.49	0.506	0.391	0.43	0.367	0.39	0									
HolPa	0.544	0.51	0.546	0.481	0.424	0.413	0.296	0								
HolCh	0.604	0.563	0.566	0.525	0.396	0.415	0.265	0.069	0							
HolPe	0.621	0.596	0.595	0.531	0.436	0.407	0.265	0.078	0.041	0						
HolJa	0.592	0.56	0.588	0.469	0.415	0.413	0.296	0.07	0.052	0.068	0					
HolAr	0.553	0.527	0.555	0.466	0.375	0.367	0.263	0.051	0.058	0.056	0.065	0				
HolBo	0.599	0.58	0.54	0.494	0.467	0.479	0.246	0.125	0.101	0.111	0.117	0.109	0			
MorSp	0.674	0.724	0.697	0.646	0.476	0.637	0.675	0.783	0.81	0.788	0.778	0.735	0.718	0		
NeBrPe	0.453	0.407	0.305	0.496	0.696	0.767	0.405	0.542	0.552	0.596	0.57	0.553	0.499	0.919	0	
NelBo	0.523	0.452	0.323	0.563	0.686	0.764	0.591	0.766	0.806	0.851	0.823	0.807	0.777	0.799	0.336	0
